# Unexpected consequences of community-based integrated care services reform: increased hospitalization expenses among older adults in China, 2011–2018

**DOI:** 10.3389/fpubh.2026.1743279

**Published:** 2026-03-18

**Authors:** Jing Xin, Li Han

**Affiliations:** 1 School of Public Administration, Zhongnan University of Economics and Law, Wuhan, China; 2 School of Law and Economics, Wuhan University of Science and Technology, Wuhan, China

**Keywords:** community-based integrated care services reform, health effects, out-of-pocket hospitalization expenses, release effect, total hospitalization expenses

## Abstract

**Introduction:**

As a result of the increasingly aging population of China, the development and implementation of effective public older adult care service strategies for older adults with nursing and medical needs have become pressing priorities. In this context, China launched a community-based integrated care services (CBICS) reform in several cities in 2016. This study evaluates the impact of the CBICS reform on hospitalization expenses among older adults.

**Methods:**

Using 2011–2018 China Health and Retirement Longitudinal Survey (CHARLS) data, we applied the difference-in-differences (DID) method to identify the causal impact of the CBICS pilot policy on the inpatient medical expenses of old adults. In addition, we robustly estimated the net effect of the CBICS reform on hospitalization expenses among older adults by combining propensity score matching (PSM) with DID while excluding other policy disturbances and adjusting the sample size.

**Results:**

Following CBICS implementation, the total hospitalization expenses and out-of-pocket hospitalization expenses increased significantly among older adults, rising by approximately CNY 1,971 and CNY 1,096, respectively. Further analysis revealed that the substantial increase in hospitalization expenses was concentrated primarily in primary healthcare facilities, and the increase in higher-level medical institutions was not statistically significant. The structural impact of the CBICS policy on inpatient cost growth offers a plausible explanation for this observed increase in medical spending. Additionally, we identify health and release effects: improved health outcomes among older adults increase the use of medical services, which, in turn, contributes to higher medical expenses. Moreover, the CBICS policy had more pronounced effects on the rural-dwelling, older, and disabled subgroups.

**Conclusion:**

The increase in hospitalization expenses for older adults resulting from the CBICS pilot policy was essentially driven by the release of medical demand that had been previously suppressed. Therefore, if the CBICS reform is aimed at simultaneously achieving both health improvement and cost control, a hierarchical medical system with two-way linkage between community and hospital should be established in the course of policy promotion. In addition, in the promotion of CBICS policy, priority must be given to the accessibility and equity of services to ensure timely access to services for vulnerable groups.

## Introduction

The provision of community-based integrated care can effectively address multiple challenges, such as high medical expenditures and the rising demand for quality care, for people with constrained healthcare budgets (1–6). Globally, many countries have implemented diverse integrated care models tailored to the characteristics of their own health systems (4, 6). Typical practices include health maintenance organizations (HMOs), such as Kaiser Permanente, in the USA (7); Integrated Care Systems (ICS) in the UK (8); and Regional Health System (RHS) programs in Singapore (9), aimed at better controlling the growth of medical expenditures, reducing unnecessary hospitalizations, and improving the quality of healthcare services for the elderly population (1, 10, 11).

However, owing to differences in socioeconomic environments, policy settings, and implementation processes across countries, the literature has not yet reached a consistent conclusion regarding the impact of CBICS on medical expenditures. For example, in developed countries such as the United Kingdom, the United States, and Canada, studies have shown that integrated care reduces healthcare expenses (10, 12–14). In contrast, some studies indicate that the implementation of CBICS pilot policies can lead to an increase rather than a decrease in medical expenditures (14, 15).

As with developed countries, China faces the challenges of high healthcare expenditure and a growing demand for care (16). Against this backdrop, in recent years, China has attempted to use CBICS as a means of improving care quality and integrating health resources. Since 2016, China has launched CBICS reform pilots. According to data from the Ministry of Civil Affairs of China, by 2020, approximately $5 billion had been invested in the development of CBICS. Currently, the CBICS is an important model for the provision of care services for older adults in China. Several studies have investigated the impact of CBICS on inpatient medical expenditures among older adults in China. For example, using CHARLS (2018) data, Sun et al. (17) reported that CBICS significantly increased the utilization of outpatient services, reduced the probability of hospitalization, and decreased total hospitalization expenses, providing strong evidence for the potential of CBICS to reduce medical expenditures. Similarly, using administrative claim data from Shenzhen, China, Ye and Jiang (18) adopted a DID model to explore the impact of the pilot integrated care model in Shenzhen and reported that the integrated care model helped to reduce hospitalization expenses for older adults. In contrast, another retrospective cohort study using health insurance utilization data from a town in Zhejiang Province, China, revealed that after the integrated care model had been established, the average hospitalization expenses increased across all levels of medical institutions (15).

Nevertheless, the existing evidence is limited in several ways. First, the study conclusions are inconsistent: some studies report an increase in hospitalization expenses (15), whereas others report a downward trend in hospitalization expenses (18). Second, few studies have explored the structural reasons underlying the impact of CBICS pilots on inpatient medical expenditures, which may limit the precision of policy optimization and thus the achievement of health equity goals. Third, some studies discuss only specific populations or use data from small geographic areas (15, 19, 20), thereby limiting the generalizability of the findings.

Therefore, in this study, longitudinal CHARLS data from 2011 to 2018 is used to investigate the impact of CBICS pilot reforms on inpatient medical expenses among older adults. CBICS can affect the inpatient medical expenses of older adults through multiple channels. The first channel is the health effect. Regular health assessments provided by the pilot program facilitate early detection and the timely treatment of diseases, which can improve the physical and mental health of older adults, thereby reducing their inpatient medical expenses (21, 22). The second channel is the risk perception effect and the moral hazard effect. During the pilot process, each pilot city promoted knowledge on disease prevention and rehabilitation, thereby enabling older adults to perceive the potential health risks that they could face in the future and promoting improvements in their health behaviors (23). However, with an increase in health information, older adults may seek medical care more frequently, thereby increasing service utilization and creating a demand-side moral hazard, which leads to the waste of medical resources (24–26). The third channel is the release effect. The CBICS reform integrates medical resources into families and communities to increase the accessibility of medical services. Improved service accessibility can stimulate demand among previously underserved people, thereby leading to an increase in overall medical service volume and medical expenses (14).

We applied the DID approach to identify the causal effects of the CBICS pilot policy on hospitalization expenses for older adults. First, we found that the CBICS pilot significantly increased total hospitalization expenses and out-of-pocket hospitalization expenses for all older adults. Second, we compared the heterogeneity of hospitalization expenses for older adults across different levels of medical institutions after the initiation of the CBICS pilot and found that the increase in hospitalization expenses was concentrated mainly in primary medical institutions, which initially proves the existence of the release effect. Third, we compared the heterogeneity among older adults with different individual characteristics and found that these heterogeneities were concentrated mainly among older adults who were advanced in age, disabled, or living in rural areas. Fourth, we found that the CBICS pilot policy increased hospitalization expenses for older adults through the release effect and health effect.

This study makes the following contributions to the literature. First, using four waves of longitudinal data covering the period of 2011 to 2018, we identify the structural reasons underlying the impact of the CBICS pilot policy on hospitalization expenses among older adults in China. Second, building on previous research, we explore the heterogeneous effects of CBICS pilots on older adults with different individual characteristics and provide empirical evidence to support the existence of the release effect. Third, we suggest another possible impact mechanism: the implementation of the CBICS pilot policy improves the physical and mental health of older adults, thereby increasing their hospitalization expenses.

## Background

### The CBICS pilot programs in China

The CBICS pilot was initiated in 2016. By 2020, five batches of cities had joined, totaling 203 pilot cities. The central government had invested approximately 5 billion yuan and triggered more than 18 billion in investments from local governments and at least 13 billion in investments from social sources, resulting in a total investment of more than 36 billion yuan.

The first two batches were announced in November 2016 and November 2017; thus, their treatment years are set as 2017 and 2018. The third batch was announced in May 2018, but since the CHARLS data were collected in the summer of 2018—shortly after the announcement—the third batch likely had no immediate effect. Thus, its treatment year is set to 2019. In this study, the treatment group includes only the first two batches; the control group includes the third, fourth, and fifth batches as well as nonpilot cities.

### Theoretical framework

#### Theoretical analysis of the impact of CBICS on the hospitalization expenses of older adults

Andersen’s behavioral model of health service use (BMHSU) is widely applied in the field of health services to analyze the factors that influence individual health service behavior (27). In this model, the factors influencing medical service utilization are divided into three categories: predisposing factors, enabling factors and need factors. Predisposing factors represent the basic characteristics of an individual, which include age, sex, education level, and marital status and drive the potential trends of medical service demand. Enabling factors are the resources endowed in the individual or environment that can promote or hinder access to medical services, including economic income, medical insurance, accessibility of medical services, and public policy support, which serve as key bridges connecting needs to utilization. Need factors are the perceived or actual health problems of individuals, and they include self-rated health status, the prevalence of chronic diseases, and the degree of disability, which are the direct factors that drive medical service utilization.

The CBICS pilot policy is a type of exogenous policy shock that belongs among enabling factors, in which medical services (preventive care, treatment, rehabilitation and end-of-life care) are integrated with core long-term care functions (such as personal assistance, daily support and psychosocial care). The pilot policy both meets current health and long-term care needs and can release unmet needs by improving accessibility, thereby affecting health behaviors, healthcare utilization and health outcomes, which can affect medical expenses. However, the direction and scale of these impacts are still uncertain because the CBICS pilot policy may influence hospitalization expenses through multiple pathways.

First, the CBICS reform may improve the health conditions of older adults, leading to an increase or decrease in medical expenses. During the pilot process, most pilot cities provided regular health assessments of the older population. In the short term, this could result in the early detection and timely treatment of diseases, which would increase the frequency of healthcare utilization and expenses. In the long term, regular health assessments can help healthcare providers play the role of gatekeepers and maintain good health (24, 28) among the older population, which can prevent the progression of diseases and reduce the cost of treatments, thereby reducing hospitalization expenses (29).

Second, the CBICS reform may entail a risk perception effect, which could influence health behaviors and potentially affect medical expenses. During the pilot process, the pilot cities disseminated knowledge regarding disease prevention and rehabilitation (23). On the one hand, this helped to improve the health literacy of older adults, thereby reducing hospitalization expenses; on the other hand, greater awareness could enhance the perception of health needs. As their levels of health information increase, older adults may seek medical care more frequently (12), increase their service utilization, and possibly experience an increase in expenses.

Third, the CBICS reform integrates medical care and older care and embeds medical resources into families and communities to improve the accessibility of medical services. This can lead to a demand-side moral hazard, that is, to a reduction of out-of-pocket expenses for older adults and of the marginal price of medical services, resulting in the overuse of services (25, 26, 30, 31). However, in rural areas prior to the reform, owing to incomplete medical service facilities and other reasons, older adults with advanced age and disabilities faced obstacles in their access to medical treatment, thus their normal health needs could not be met. After the reform, pilot cities provided services such as home care and health consultation, which both enhanced primary medical services and improved the accessibility of medical services. The removal of these obstacles immediately addresses long-term unmet health needs, thereby increasing the usage and costs of services (14).

Therefore, changes in the medical expenses of older adults are shaped by four potential mechanisms: the “health effect,” the “risk perception effect,” the “moral hazard effect,” and the “release effect.” These mechanisms operate through the three key pathways of health status, health behavior, and the utilization of medical services. The CBICS reform affects these factors, ultimately influencing the hospitalization expenses of older adults. Given the complex interactions among these influences, the following hypotheses are proposed:

*H1A*: The CBICS reform reduces the hospitalization expenses of older adults.

*H1B*: The CBICS reform increases the hospitalization expenses of older adults.

*H2*: The CBICS reform affects the hospitalization expenses of older adults through changes in their health status, healthcare behavior, and utilization of medical services.

#### Heterogeneous effects of the CBICS reform on hospitalization expenses among older adults

According to the Andersen model, the impact of the CBICS policy on older adults’ hospitalization expenses is heterogeneous because of the differences in individuals’ predisposing characteristics and need factors, which is reflected in three aspects. The first is the urban–rural disparity. In China, the accessibility of healthcare services is better in urban areas than it is in rural areas (32). This gap implies that urban and rural older adults may receive different benefits from the CBICS system. The second is age disparity. As people age, they experience poorer health conditions and higher care needs. This leads to differences in their uses and costs of medical services, which suggests that the CBICS policy may have different effects on different age groups. is the third is health status disparity. People seek healthcare to improve their health conditions rather than simply to obtain services. Therefore, the CBICS policy may have different effects on older adults with better health conditions than it does on those with poorer health conditions.

*H3*: The effects of the CBICS reform on hospitalization expenses among older adults vary by subgroup.

## Materials and methods

### Data source

We used data from the China Health and Retirement Longitudinal Survey (CHARLS) covering the years of 2011, 2013, 2015 and 2018. CHARLS is a large-scale national interdisciplinary follow-up survey project led by the National Institute of Development of Peking University. CHARLS collected comprehensive information on personal characteristics, economic status, health status and medical service utilization among Chinese middle-aged and older adults over 45 years and above. Using a stratified random sampling method to select the sample, a national baseline survey was conducted for CHARLS in 2011. The sample was very representative, including 17,708 respondents from 10,257 households in 28 provinces (autonomous regions and municipalities) in China. Subsequently, CHARLS follow-up surveys were conducted in 2013, 2015, 2018 and 2020, and its sample also increased to 198.17 million respondents from 12,400 households. The reasons for selecting the CHARLS database as the source of micro data for this study are as follows: First, CBICS primarily targets older individuals, and CHARLS was specifically focused on adults over 45 years and older, which ensures population relevance. Second, this study examines the impact of CBICS on hospitalization medical expenses, and CHARLS contains detailed questions on hospitalization expenses, which provides direct empirical support. Third, the data cover 125 prefecture-level cities, including 17 from the first batch and 9 from the second batch of CBICS pilot programs, which were both fully captured within the 2011–2018 period. Therefore, the treatment group in this analysis includes only the first two pilot batches, while later batches are used as part of the control group.

### Study sample

The study sample comprised older adults 60 years of age or older. In this study, we excluded people under 60 years of age and those with missing information, and the final study sample was an unbalanced panel dataset consisting of 17,484 observations from 125 prefect-level municipalities.

### Variables

The outcome variable used in this study is hospitalization expenses. Consistent with previous research (32, 33), we measure hospitalization expenses using two variables: total hospitalization costs and out-of-pocket hospitalization expenses. In the CHARLS questionnaire, respondents reported the total hospitalization expenses and out-of-pocket portion for all their hospitalizations over the previous year. All of the cost variables in this study have been adjusted to 2011 Chinese yuan (CNY) using the consumer price index (CPI).

The key independent variable is the implementation status of CBICS policy. The interaction term $T_{\mathrm{ij}}\times D_{t}$ is assigned a value of 1 if city j where older adult i resides implemented the CBICS policy in year t and 0 otherwise.

The control variables include age, sex (male = 1, female = 0), educational attainment (junior high school or above = 1, below junior high school = 0), marital status (married and living with spouse = 1, other = 0), household registration (rural = 1, nonrural = 0), public health insurance coverage (covered = 1, not covered = 0), annual household income (log-transformed), number of children, retirement status (retired = 1, employed = 0), objective health status as represented by ADL score, and subjective health status as represented by a self-rated health score.

### Empirical strategies

The identification strategy used for this study is the adoption of a difference-in-differences (DID) model to identify whether the implementation of CBICS affects the hospitalization medical expenses of older adults. The DID model is a quasiexperimental method widely used for policy evaluation and causal inference. Its core goal is to estimate the net causal effect of a policy or intervention by comparing changes in outcomes between a treatment group (affected by the policy) and a control group (unaffected by the policy) before and after the intervention (34). The basic regression model is as follows:


$$Y_{\mathrm{ijt}}=\alpha_{0}+\beta T_{\mathrm{ij}}\times D_{t}+\theta_{t}+X_{\mathrm{ijt}}+A_{i}+\varepsilon_{\mathrm{ijt}}$$ (1)

where $Y_{\mathrm{ijt}}$ represents a group of individual-level outcome variables. Two indicators are adopted in this study: total hospitalization medical expenses in the previous year and total out-of-pocket hospitalization medical expenses in the previous year. These indicators can be used to identify the medical burden of individuals. $T_{\mathrm{ij}}$ represents whether individual i is in a pilot city in j . If the city where individual i is located was a pilot city in 2018, then individual i is defined as belonging to the treatment group; that is, $T_{\mathrm{ij}}$ = 1; otherwise, individual i belongs to the control group ( $T_{\mathrm{ij}}$ = 0). $T_{\mathrm{ij}}\times D_{t}$ is the interaction term between the dummy variable of the pilot cities in the treatment group and the dummy variable before and after the implementation of the CBICS reform. The coefficient β of the interaction term is the core coefficient of concern in this study, and it represents the difference in the outcome variable of the older adult population in the pilot cities resulting from the implementation of CBICS compared with that in other regions. Accordingly, the policy effect of the CBICS reform is examined, with a specific focus on whether the CBICS policy affects the hospitalization medical expenses of older adults. $A_{i}$ denotes the individual fixed effects, $\theta_{t}$ indicates the time fixed effects, and $X_{\mathrm{it}}$ stands for a set of individual-level control variables, including age, sex, education level, marital status, household registration, medical insurance, annual earned income (logarithm), number of children, retirement status, the ADL variable representing objective health status, and self-reported health representing subjective health status. $\varepsilon_{\mathrm{ijt}}$ is the disturbance term.

In Model 1, the treatment and control groups are assumed to follow parallel trends without the policy. Since the counterfactual—how the treatment group would change without intervention—cannot be observed, prepolicy trends are examined. If older adults’ hospitalization expenses in pilot and nonpilot cities followed similar paths prior to policy implementation, then the assumption is supported. Two methods are used to test this: trend plotting and event study models. Trend plotting relies on visual inspection, which is subjective and not statistically rigorous. The event study method uses interaction terms between year dummies and the treatment dummy in a regression. The coefficients indicate the differences between the groups each year, which results in a more accurate and robust test. In this research, the event study approach was used to test parallel trends before and after implementation. Model 2 is specified as follows:


$$Y_{\mathrm{ijt}}=\alpha_{0}+\sum_{t=2011}^{2018}\beta_{t}(T_{\mathrm{ij}}\times D_{t})+\theta_{t}+X_{\mathrm{ijt}}+A_{i}+\varepsilon_{\mathrm{ijt}}$$ (2)

where $\beta_{t}$ identifies the dynamic changes in the inpatient medical expenses of the treatment group samples relative to those of the control group samples over the subsequent three years, with 2015 used as the base group. The settings of the other parameters are consistent with those in Equation 1.

## Results

### Descriptive statistics

Table 1 presents the descriptive statistics for the full sample, treatment group, and control group and the pairwise comparison results between the treatment group and the control group before and after the implementation of home and community older adult care services. After pilot policy implementation, the total hospitalization expenses of the older adults in the treatment group decreased compared with those prior to policy implementation, but the out-of-pocket hospitalization expenses increased compared with those prior to policy implementation. However, for the control group samples, both the total hospitalization expenses and the out-of-pocket hospitalization expenses of older adults increased following policy implementation. After policy implementation, the disability levels of both the treatment group and the control group decreased, and the self-rated health level increased. These findings offer an initial indication that the reform pilot of CBICS has played a certain role in improving the physical health of older adults.

**Table 1:** Descriptive statistics.

Variables	Full		Post = 0		Post = 1	
	Mean	Sd.	Treatment	Control	Treatment	Control
Total hospitalization expenses	1032.363	9402.467	2459.921	873.562	2175.529	1431.162
Out-of-pocket hospitalization expenses	391.023	5444.922	371.485	272.593	927.13	746.129
ADL	1431.162	1431.162	0.79	0.72	0.308	0.523
Number of surviving children	746.129	746.129	2.553	2.642	1.795	2.933
Retirement (=1)	0.139	0.355	0.323	0.13	0.154	0.049
Age group	5.637	1.368	5.734	5.636	5.41	5.453
Male (=1)	0.529	0.499	0.547	0.529	0.359	0.481
Education level	1.785	1.12	1.957	1.759	2.077	2.477
Self-reported health	2.713	0.764	2.707	2.708	3.256	2.886
Public health insurance (=1)	0.74	0.438	0.735	0.736	0.974	0.926
Married (=1)	0.623	0.485	0.578	0.624	0.744	0.686
Rural hukou (=1)	0.829	0.377	0.67	0.839	0.949	0.747
Personal annual income (log)	4.663	4.193	5.956	4.513	8.403	7.591

Age group assignments are as follows: 45–49 = 1; 50–54 years = 2; 55–59 years = 3; 60–64 years = 4; 65–69 years = 5; 70–74 years = 6; 75–79 years = 7; and 80 and above = 8. Education level group assignments are as follows: a junior high school degree or above is set as 1, whereas a junior high school degree or below is set as 0.

### Baseline results

Table 2 presents the estimated impact of the CBICS policy on the hospitalization medical expenses of older adults. Columns (1) and (3) show the regression results with only the explanatory variables included, while columns (2) and (4) present the overall results with all of the variables included. In the regression process, individual and time fixed effects were controlled for.

**Table 2:** Impact of CBICS on hospitalization expenses.

Variables	(1)	(2)	(3)	(4)
	Total hospitalization expenses	Total hospitalization expenses	Out-of-pocket hospitalization expenses	Out-of-pocket hospitalization expenses
Panel A. All Healthcare Institutions				
$T_{\mathrm{ij}}\times D_{t}$	2218.4296***	1970.8962***	1125.9776***	1096.4943***
	(4.03)	(3.58)	(3.43)	(3.12)
Constant	987.0116***	4381.3051*	251.7223***	1382.3382
	(2393.04)	(1.88)	(1028.92)	(1.18)
Observations	17,362	17,362	17,449	17,449
Panel B. Primary Healthcare Institutions				
$T_{\mathrm{ij}}\times D_{t}$	2256.9791***	1952.7875***	1531.9218***	1467.4982***
	(4.10)	(3.39)	(4.13)	(3.64)
Constant	958.7461***	5276.3228**	234.6578***	1398.1511
	(2232.84)	(2.14)	(815.44)	(1.15)
Observations	16,671	16,671	16,752	16,752
Panel C. Higher-Level Healthcare Institutions				
$T_{\mathrm{ij}}\times D_{t}$	−6271.6218	4640.8322	−4848.7418	−5287.8135
	(−1.44)	(0.58)	(−1.05)	(−1.01)
Constant	7192.4556***	13874.5005	1277.6199***	20430.8135
	(2598.63)	(0.36)	(447.78)	(1.17)
Observations	1,570	1,570	1,620	1,620
Control variables	No	Yes	No	Yes
ID FE	Yes	Yes	Yes	Yes
Year FE	Yes	Yes	Yes	Yes

Robust standard errors are reported in parentheses. ***, **, and * indicate significance levels of 1, 5, and 10%, respectively. Each regression controls for individual covariates, city-level covariates, individual FEs, and year FEs. The individual covariates include age, sex, education level, marital status, household registration status, public health insurance status, annual income (logarithm), number of children, retirement status, ADLs, and self-reported health.

Panel A of Table 2 presents the results using the full sample. Columns (1) and (2) show that the CBICS reform pilot significantly increased the total hospitalization medical expenses of older adults. Column (2) indicates that the CBICS pilot increased the total hospitalization medical expenses of the older adult population by CNY 1,970.896 (this result is significant at the 1% level). Columns (3) and (4) show that the CBICS pilot significantly increased the out-of-pocket hospitalization medical expenses of older adults. Column (4) indicates that the CBICS policy increased the out-of-pocket hospitalization medical expenses of older adults by CNY 1,096.494 (this result is significant at the 1% level). Hypothesis 1B is therefore verified.

Panels B and C of Table 2 report the estimates for primary care institutions and higher-level medical institutions, respectively. In Panel B, the increase in medical expenses due to the CBICS pilot program is concentrated in primary care institutions. The data displayed in columns (2) and (4) show that inclusion in CBICS coverage leads to increases of CNY 1952.788 and CNY 1467.4982 in total hospitalization expenses and out-of-pocket hospitalization expenses for older adults, respectively (these results are significant at the 1% level). The regression results reported in Panel C indicate that in higher-level medical institutions, the effect of the CBICS pilot program on the increase in medical expenses is nonsignificant. These findings indicate that in contexts where the demand for care and even medical services has been suppressed for a long time, the implementation of CBICS has a demand-releasing effect and can effectively regulate the allocation and utilization of medical resources, which is conducive to the optimal allocation of the entire medical service industry.

### Robustness

**Figure 1:**
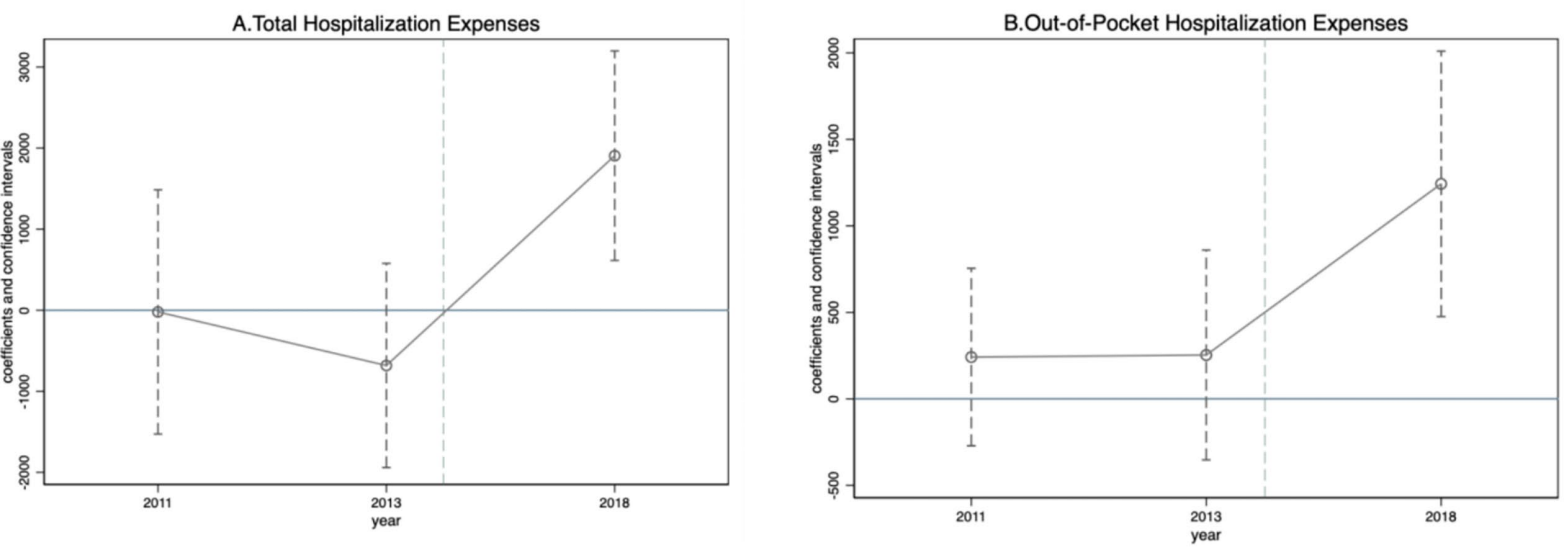
Parallel trend tests. The CBICS policy was implemented in 11 pilot cities in either 2016 or 2017. On the x-axis, the year 2015 is omitted because it is treated as the benchmark year. All regressions control for individual covariates, individual fixed effects, and year fixed effects. The individual-level covariates are consistent with those presented in Table 2 **(A)** Total hospitalization expenses. **(B)** Out-of-pocket hospitalization expenses.

We first tested the parallel trend assumption. A crucial prerequisite for applying the DID method is meeting the parallel trend assumption, which, in this case, means that if there were no reform pilot programs, then the trends in the changes in individuals’ inpatient medical expenses in pilot and nonpilot cities should be parallel. To test this possibility,following the approach of Currie et al. (23), we conducted a parallel trend test by incorporating interaction terms between the pilot city dummy and year dummy variables into the regression, as specified in Equation 2. Using 2015 as the reference group, the results are shown in Figure 1. Prior to 2015, there was no significant change in the differences in total inpatient medical expenses or annual out-of-pocket inpatient medical expenses for older adults between pilot cities and nonpilot cities. Following policy implementation, the average annual inpatient expenses and annual out-of-pocket inpatient expenses for older adults in pilot cities significantly increased, which indicates that the DID model in this study satisfies the parallel trend assumption and that the benchmark regression results reported in Table 2 are reliable.

Second, to address the endogeneity that arises from the nonrandom implementation of policies and to mitigate potential selection bias, we used the PSM-DID approach to reestimate the effect of the CBICS policy on older adults’ medical expenses. This procedure involved two steps. First, a logit model was used to estimate the propensity scores, or the probability of a city being selected as a pilot for the CBICS reform. Then, for each pilot city (treatment group), nonpilot cities with similar propensity scores were identified and matched using a 1:2 nearest neighbor matching algorithm. The results of the PSM are presented in Table 3. Second, Model 1 was applied to the matched sample to estimate the policy’s impact on the medical expenses of older adults. The PSM-DID estimation results reported in Panel A of Table 4 indicate that the coefficient on the interaction term remains statistically significant and closely aligned with the benchmark regression, thereby confirming the robustness of the findings.

**Table 3:** Results of the PSM for the treatment and control groups.

Variables	Unmatched (U)	Mean		% Bias	% Reduct	t Test	*p* value
	Matched (M)	Treated	Control		|bias|	*t*	
ADL	U	0.73417	0 0.70693	2.0		0.65	0.514
	M	0.73417	0 0.7001	2.5	−25.1	0.56	0.576
Number of surviving children	U	2.547	2.6594	−4.7		−1.44	0.149
	M	2.547	3.1502	−25.4	−436.7	−5.65	0.000
Retirement (=1)	U	0.31766	0.12791	45.7		16.94	0.000
	M	0.31766	0.31958	−0.5	99.0	−0.09	0.928
Age group	U	5.7063	5.6264	5.8		1.84	0.066
	M	5.7063	5.5931	8.2	−41.6	1.84	0.066
Male (=1)	U	0.53839	0 0.52719	2.2		0.70	0.481
	M	0 0.53839	0 0.53023	1.6	27.2	0.37	0.709
Education level	U	1.9607	1.7758	15.7		5.20	0.000
	M	1.9607	1.857	8.8	43.9	1.90	0.057
Self-reported health	U	2.7342	2.7152	2.5		0.78	0.436
	M	2.7342	2.7917	−7.5	−204.3	−1.71	0.088
Public health insurance (=1)	U	0.73992	0 0.74024	−0.1		−0.02	0.982
	M	0.73992	0.68138	13.3	−18333.4	2.95	0.003
Married (=1)	U	0.58541	0.62605	−8.3		−2.63	0.008
	M	0.58541	0.60221	−3.4	58.7	−0.78	0.435
Rural hukou (=1)	U	0.67946	0.83781	−37.6		−13.28	0.000
	M	0.67946	0 0.6761	0.8	97.9	0.16	0.870
Personal annual income (log)	U	6.0625	4.5849	36.0		11.11	0.000
	M	6.0625	5.9333	3.1	91.3	0.73	0.467

**Table 4:** Robustness test.

Variables	(1)	(2)
	Total hospitalization expenses	Out-of-pocket hospitalization expenses
Panel A. PSM-DID		
$T_{\mathrm{ij}}\times D_{t}$	1932.039***	1072.803***
	(551.00)	(351.97)
Observations	16,821	16,821
Panel B. Adjusting the sample size		
Elimination of those municipalities directly under the Central Government		
$T_{\mathrm{ij}}\times D_{t}$	1970.896***	1096.494***
	(550.98)	(351.19)
Observations	17,362	17,449
Exclusion of the third batch of pilot cities		
$T_{\mathrm{ij}}\times D_{t}$	2002.475***	1110.606***
	(546.64)	(352.33)
Observations	16,023	16,109
Panel C. Exclusion of other policy interference		
Pilot projects that integrate medical care with elderly care	2059.0533**	857.7531*
	(2.13)	(1.68)
Observations	17,362	17,449
Pilot long-term care insurance	1972.8208***	1072.7420***
	(3.44)	(2.95)
Observations	17,362	17,449
simultaneous exclusion of the combination of medical care and long-term care insurance policies	2059.0519**	857.7459*
	(2.13)	(1.68)
Observations	17,362	17,449
Panel D. Treating the dependent variable with log transformation		
$T_{\mathrm{ij}}\times D_{t}$	0.6901***	0.6525***
	(4.99)	(5.30)
Observations	17,484	17,484
Control variables	YES	YES
ID FE	YES	YES
Year FE	YES	YES

Robust standard errors are reported in parentheses. ***, **, and * indicate significance levels of 1, 5, and 10%, respectively. Each regression controls for individual covariates, city-level covariates, individual FEs, and year FEs. The individual covariates include age, sex, education level, marital status, household registration status, public health insurance status, annual income (logarithm), number of children, retirement status, ADLs, and self-reported health.

Third, the sample size was adjusted. We first deleted those municipalities directly under the Central Government. During the pilot implementation, these municipalities reported at the district level. If one district was selected as a pilot, then the entire municipality was classified as a pilot city, which can potentially dilutes the estimated policy effect because of partial coverage. To address this measurement inconsistency, these municipalities were excluded from the data reported in Panel B of Table 4. The results confirm that the positive effect of the pilot policy on hospitalization medical expenses for older adults remains statistically significant and robust. We next excluded the third batch of pilot cities. Although the third batch was officially announced in 2018, its implementation coincided closely with the CHARLS 2018 survey period, raising concerns about whether there was sufficient time for the policy effects to materialize. Therefore, the third batch of cities was also excluded in the data reported in Panel B of Table 4. The findings continue to show a robust positive impact, which reinforces the reliability of the baseline estimates.

Fourth, other policy disturbances were excluded. Previous studies have shown that both long-term care insurance (LTCI) pilots and integrated medical and social care policy (IMSCP) pilots affect the medical expenses of older adults (21, 35). To eliminate the potential influence of these two types of pilot policies, the interaction terms of the dummy variables for the LTCI pilot regions and the IMSCP pilot regions were simultaneously added with the implementation time points of their respective policies to the benchmark regression detailed in Table 4. The results displayed in Panel C of Table 4 show that the CBICS reform pilot still significantly increased inpatient medical expenses for older adults after controlling for the interference of other policies. Therefore, a benchmark regression that does not exclude the interference of these two types of policies should not lead significantly biased results.

Fifth, we applied logarithmic transformation to the explained variable. Since hospitalization expenses typically have an asymmetric distribution, logarithmic transformation can effectively reduce the impact of extreme values and increase the normality of the data distribution. The results displayed in Panel D of Table 4 show that after logarithmic transformation, the CBICS pilot reform increased total hospitalization expenses and out-of-pocket hospitalization expenses for older adults by 69 and 65%, respectively. The estimated coefficients are all significant at the 1% level, which further supports the robustness of the research conclusions.

### Heterogeneity

On the basis of the research conclusions described in the previous section, we further examine the heterogeneous effects of CBICS policy on different demographic groups to more comprehensively evaluate its effects.

Age heterogeneity: older adults of advanced age often suffer from multiple chronic diseases, face functional decline, and have a high risk of disability, which makes them a high-demand group for medical services. Healthcare services provided by CBICS, such as 24-h health monitoring, can promptly detect health abnormalities and meet the medical service needs of older adults of advanced age in a timely manner, thereby increasing their hospitalization expenses. As shown in columns (1) to (4) of Table 5, CBICS increases the total hospitalization expenses and out-of-pocket hospitalization expenses of older adults of advanced age, but the impact on older adults who are not of advanced age is nonsignificant. The effect of the CBICS policy on hospitalization expenses among older adults of advanced age is significant at the 1% level. For every additional unit of home- and community-based older adult care service supply, the total hospitalization expenses of older adults of advanced age increase by approximately CNY 5,732, and their out-of-pocket hospitalization expenses increase by approximately CNY 4,044. These findings are similar to the results obtained by Leutz et al. (36), who reported that as the proportion of people over 85 years and above among Social Health Maintenance Organization (SHMO) members increases, the cost of HCB care benefits also increases. This outcome indirectly suggests that older adults of advanced age have a higher service demand and utilization intensity of healthcare services.

**Table 5:** Heterogeneity in age.

Variables	Total hospitalization expenses		Out-of-pocket hospitalization expenses	
	Age> = 70	Age<70	Age> = 70	Age<70
	(1)	(2)	(3)	(4)
$T_{\mathrm{ij}}\times D_{t}$	5732.7405***	−2580.5754*	4044.8232***	−1469.2839
	(6.67)	(−1.81)	(17.87)	(−1.44)
Constant	3171.5971*	3083.5310**	1116.3221	639.5154
	(1.92)	(2.35)	(1.46)	(1.33)
Control variables	Yes	Yes	Yes	Yes
ID FE	Yes	Yes	Yes	Yes
Year FE	Yes	Yes	Yes	Yes
Observations	8,679	8,264	8,749	8,282
R-Squared	0.761	0.699	0.297	0.666

Robust standard errors are reported in parentheses. ***, **, and * indicate significance levels of 1, 5, and 10%, respectively. Each regression controls for individual covariates, city-level covariates, individual FEs, and year FEs. The individual covariates include age, sex, education level, marital status, household registration status, public health insurance status, annual income (logarithm), number of children, retirement status, ADLs, and self-reported health.

Health heterogeneity: Disabled older adults face severe limitations in their daily living activities and are highly dependent on care and medical services. In China, families are the primary source of informal care and play a central role in supporting this population. CBICS provide professional medical and health services that complement family caregiving, and family caregivers have a strong preference for these services (37). As shown in columns (1)–(4) of Table 6, CBICS significantly increases both the total and out-of-pocket hospitalization expenses for disabled older adults, and the effects are significant at the 1% level. Each additional unit of CBICS supply is associated with an increase of approximately CNY 7,581 in total hospitalization expenses and that of CNY 5,074 in out-of-pocket expenses. These elevated hospitalization expenses likely reflect the fulfillment of previously unmet and urgent healthcare and long-term care needs among the households of disabled older adults.

**Table 6:** Heterogeneity in health status.

Variables	Total hospitalization expenses		Out-of-pocket hospitalization expenses	
	Not disabled	Disabled	Not disabled	Disabled
	(1)	(2)	(3)	(4)
$T_{\mathrm{ij}}\times D_{t}$	−31.9198	7581.4337***	−162.4475	5074.4853***
	(−0.04)	(3.88)	(−1.07)	(3.58)
Constant	11058.7210**	3192.9170	4126.0796	−487.4217
	(2.04)	(1.04)	(1.65)	(−0.22)
Control variables	Yes	Yes	Yes	Yes
ID FE	Yes	Yes	Yes	Yes
Year FE	Yes	Yes	Yes	Yes
Observations	8,372	7,664	8,391	7,737
R-Squared	0.754	0.824	0.597	0.463

Robust standard errors are reported in parentheses. ***, **, and * indicate significance levels of 1, 5, and 10%, respectively. Each regression controls for individual covariates, city-level covariates, individual FEs, and year FEs. The individual covariates include age, sex, education level, marital status, household registration status, public health insurance status, annual income (logarithm), number of children, retirement status, ADLs, and self-reported health.

Residential heterogeneity: The urban–rural gap in China has led to significant differences among rural older adults in various aspects, including poorer health conditions and a higher incidence of depression. Moreover, most rural older adults rely entirely on basic public pensions, and rural areas face challenges in the provision of care services, such as limited resources for specialized medical services and high-quality care, which has long suppressed their medical needs. Eliminating the urban–rural gap in medical service supply and promoting resource decentralization are important goals of CBICS (38). Therefore, there should be no urban–rural disparity in CBICS or the medical service supply. The estimation results reported in columns (1)–(4) of Table 7 show that CBICS increases the total hospitalization expenses and out-of-pocket hospitalization expenses of rural residents, and this effect is significant at the 1% level. These findings suggest that the implementation of the CBICS reform enhances the service capacity and accessibility of primary medical institutions, which has a more significant impact on rural older adults.

**Table 7:** Heterogeneity in place of residence.

Variables	Total hospitalization expenses		Out-of-pocket hospitalization expenses	
	City	Country	City	Country
	(1)	(2)	(3)	(4)
$T_{\mathrm{ij}}\times D_{t}$	−3079.1145	1765.4282***	−2467.0111**	1020.3438***
	(−1.65)	(3.04)	(−2.15)	(2.65)
Constant	2045.8459	4572.8003*	1378.8762**	1341.5216
	(1.03)	(1.70)	(2.53)	(0.93)
Control variables	Yes	Yes	Yes	Yes
ID FE	Yes	Yes	Yes	Yes
Year FE	Yes	Yes	Yes	Yes
Observations	3,875	14,101	3,891	14,171
R-Squared	0.079	0.613	0.063	0.460

Robust standard errors are reported in parentheses. ***, **, and * indicate significance levels of 1, 5, and 10%, respectively. Each regression controls for individual covariates, city-level covariates, individual FEs, and year FEs. The individual covariates include age, sex, education level, marital status, household registration status, public health insurance status, annual income (logarithm), number of children, retirement status, ADLs, and self-reported health.

### Mechanisms

As discussed earlier, changes in hospitalization expenses among older adults are jointly shaped by four potential mechanisms, namely, “health effects,” “risk perception effects,” “moral hazard effects,” and “release effects,” which work through three key pathways: health status, health behavior, and health service utilization. To this end, the potential impact mechanism is empirically examined in this study. We tested the health effect by health status, where health status was measured mainly through both subjective and objective health indicators, and the subjective health indicators mainly referred to mental health. For the CHARLS data, we used the method of Liu et al. (38) to calculate the CESD score of elderly people, which ranged from 0 to 30, with lower CESD scores indicating better mental health status. In accordance with the methods of Ma et al. (35), objective health indicators were mainly measured from two aspects: the number of chronic diseases and the presence of pain. The risk perception effect was tested by health behavior, which was measured as the sum of the two dimensions of smoking and drinking, where the higher that the sum value was, the healthier the behavior. The use of medical services was used to test the moral hazard effect and release effect and was measured mainly by the length of hospitalization.

The data shown in columns (1)–(3) of Table 8 indicate that the CBICS pilot significantly improved the mental and physical health of elderly people, and these results are similar to those of Jia et al. (39), Liu et al. (38), and Polsky et al. (22), who reported similar conclusions. The results indicate that health promotion is an important mechanism through which CBICS affects the hospitalization medical expenses of older adults, and this finding is consistent with the results of the theoretical analysis above. The regression results displayed in columns (4) and (5) reveal that CBICS did not significantly improve the health behavior of elderly people, indicating that CBICS could not promote an improvement in the health literacy of elderly people. This indicates that the risk perception effect did not exist among our sample. These results are consistent with the findings of Courbage, C., & de Coulon, A. (40) and Manning WG et al. (30), who reported that exogenous health policy shocks have no significant effect on health behavior. The regression results reported in columns (6) and (7) show that CBICS implementation significantly increased the length of hospital stay and the number of hospitalizations among elderly individuals. These results are consistent with the findings of Tomita et al. (41), Jones et al. (42) and Punchik et al. (43) and indicate that medical service utilization is an important mechanism affecting hospitalization medical expenses, which is in line with the results of the theoretical analysis described above. Combined with the baseline regression and heterogeneity analysis results, the increases of hospitalization expenses among old adults resulting from CBICS were concentrated in primary medical institutions, and the increases in hospitalization expenses resulting from CBICS among elderly people were more significant for old adults of advanced age, those who were disabled and those who lived in rural areas, which further indicates the existence of a release effect. The health and release effects proposed in Hypothesis 2 were verified.

**Table 8:** Mechanism.

Variables	(1)	(2)	(3)	(4)	(5)	(6)	(7)
	CESD	Chronic diseases	Hurts	Smoking	Drinking	Inpatient Days	Inpatient times
$T_{\mathrm{ij}}\times D_{t}$	−6.733^***^	−0.166^**^	−0.153^***^	−0.1047	−0.0591	1.294^**^	1.3223***
	(0.40)	(0.08)	(0.03)	(−1.45)	(−0.18)	(0.61)	(12.05)
Constant	15.832^***^	1.998^***^	0.909^***^	−0.0209	7.2415**	3.446	1.4526**
	(2.06)	(0.54)	(0.16)	(−0.10)	(2.53)	(2.74)	(2.51)
Control variables	Yes	Yes	Yes	Yes	Yes	Yes	Yes
ID FE	Yes	Yes	Yes	Yes	Yes	Yes	Yes
Year FE	Yes	Yes	Yes	Yes	Yes	Yes	Yes
Observations	17,483	17,484	17,484	16,673	15,025	17,452	17,484
R-Squared	0.817	0.878	0.660	0.876	0.946	0.750	0.699

Robust standard errors are reported in parentheses. ***, **, and * indicate significance levels of 1, 5, and 10%, respectively. Each regression controls for individual covariates, city-level covariates, individual FEs, and year FEs. The individual covariates include age, sex, education level, marital status, household registration status, public health insurance status, annual income (logarithm), number of children, retirement status, ADLs, and self-reported health.

## Discussion

As a result of the acceleration of the aging process, governments globally are committed to providing higher-quality healthcare services for older adults. However, the contradiction between high-quality healthcare services and medical cost control is becoming increasingly severe. The development and improvement of community-based integrated healthcare systems may be a potential solution, such as Brazil’s Family Health Strategy and the Patient-Centered Medical Home (PCMH) model implemented in Australia (44, 45). Similarly, to address aging and the increasing burden of medical care, the Chinese government launched the CBICS reform in 2016. This study uses CHARLS2011-2018 data and applies a DID approach to analyze the impact of CBICS policy pilots on hospitalization expenses for old adults.

We find that the implementation of CBICS led to an increase rather than a decrease in hospitalization expenses for older adults. After the implementation of the CBICS reform, both total hospitalization expenses and out-of-pocket hospitalization expenses increased significantly for older adults, rising by approximately CNY 1,970 and CNY 1,096, respectively. Further analysis revealed that this significant increase in hospitalization expenses was concentrated mainly in primary medical institutions, whose total hospitalization expenses and out-of-pocket hospitalization expenses increased by approximately CNY 1,953 and CNY 1,467, respectively, while the increase in hospitalization expenses among higher-level medical institutions was nonsignificant. These results provide suggestive evidence that the CBICS policy may have prompted structural changes in the utilization of medical services. This is consistent with previous research on CBICS conducted in Shanghai, China. The literature indicates that the CBICS pilot may have led to a structural shift in the utilization pattern of medical services, transitioning from high-cost hospital services to low-cost CBICS services, and such a shift releases additional demand due to improved service accessibility (24). In China, the concentration of cost growth in primary care indicates that CBICS has successfully redirected medical services to more accessible settings, particularly for rural and vulnerable populations. Although short-term expenses may increase, this transition is likely to generate long-term savings by preventing avoidable hospitalizations and reducing the burden on overstretched higher-tier medical institutions.

With respect to the impact on the volume of medical services, the results show that the implementation of the CBICS reform led to a significant increase of 1.294 days in the hospital stay of older adults. Wang et al. (46) reported a similar phenomenon; that is, the use of Medicaid home and community-based services is associated with shifting post-acute care from Medicare to the home environment, even though such a shift may increase overall care utilization. These findings therefore indirectly confirm the release effect in this context.

Another potential mechanism is the association of health improvement with higher hospitalization expenses. This study reveals that CBICS coverage reduced the depression score of older adults by 6.733 points and decreased the probability of chronic disease and pain by 16.6 and 15.3%, respectively. These findings are consistent with those reported by Jia et al. (39), who found that CBICS significantly improved the mental health of older adults and reduced cognitive impairment. Valluru et al. (47) reported that CBICS increased the survival probability of frail older adults and enhanced their resistance to major health shocks.

Furthermore, this study indicates that for older disabled adults of advanced age, the CBICS policy had a more significant effect on increasing hospitalization medical expenses. These groups often have the most urgent medical needs and the poorest access to medical services. This finding reflects that CBICS policy implementation enabled these vulnerable groups to receive more medical services, thereby leading to a significant increase in their medical expenses. For older adults living in rural areas, CBICS coverage had a more significant effect on the increase in hospitalization medical expenses. This is due to insufficient funds and insufficient policy attention in underdeveloped areas, which causes imbalanced development of medical services across various regions. Since 2011, China has sought to develop community care services in both urban and rural areas. By 2018, the gap in community care services between urban and rural areas had significantly narrowed. These findings help explain why we also reported that the impact of the CBICS policy on the hospitalization medical expenses of rural older adults significantly increased, whereas that on urban older adults significantly decreased, which further confirms the existence of the “release effect” rather than the “moral hazard effect.”

Of course, this research has several limitations which should be acknowledged. First, CBICS encompasses various service types, and the effects of different service types on inpatient medical expenses may vary. Future research should place a greater focus on precisely identifying and evaluating the comparative heterogeneous effects of different service models to guide policy optimization. Second, our emphasis was on the impact of CBICS policy on the inpatient medical expenses of older adults. However, this focus was based on the perspective of patients and did not incorporate the perspective of service providers, thus future research should conduct a CBICS policy evaluation from the perspective of service providers (for example, based on CBICS financing perspective), which is of key significance for fully understanding the effect of policy implementation, identifying any unintended consequences, and designing appropriate reform strategies. Third, older adults in rural areas face unique issues in terms of their accessibility to medical resources. Although this study reveals that CBICS has a more significant impact on medical expenses in rural areas than it does in urban areas, future research should explore the corresponding implementation models, the service content of rural CBICS programs, and their specific impacts on the medical service utilization and expenses of rural older adults in further detail. Fourth, owing to data availability limitations, the dataset used in this study covers the period of 2011 to 2018, and it uses a relatively short time span before and after the implementation of CBICS. Future studies could collect both older and more recent data to assess the impact of CBICS on hospitalization expenses.

## Conclusion

This study provides empirical evidence that CBICS can improve the physical and mental health of older adults, which implies that CBICS can help achieve the healthcare goal of ensuring healthy lives for all by 2030, as proposed in the United Nations’ 2015 Sustainable Development Goals (48). In addition, the CBICS reform unleashed previously unmet needs for medical services among elderly people of advanced age, those with disabilities, and those living in rural areas. In terms of policy design, a hierarchical medical system with a two-way linkage between communities and hospitals should be constructed to promote hospital resources among primary communities, improve community-level medical service capacities, and achieve a closed loop comprising a hierarchical medical system that places minor diseases in the community, serious diseases in the hospital, and rehabilitation back with the community to curb the unreasonable increase in medical expenses at the source. Finally, in CBICS promotion, priority must be given to the availability and equity of services, the assurance of timely access to services for vulnerable groups, and the guarantee of health rights for marginalized groups, and for rural older people in particular.

## Data Availability

Publicly available datasets were analyzed in this study. This data can be found here: the datasets used in this study is from China Health and Retirement Longitudinal Survey (CHARLS), which are publicly available in the CHARLS repository (https://charls.pku.edu.cn/en).
